# Neural cue reactivity is not stronger in male than in female patients with alcohol use disorder

**DOI:** 10.3389/fnbeh.2022.1039917

**Published:** 2022-11-16

**Authors:** Sarah Gerhardt, Sabine Hoffmann, Haoye Tan, Martin Fungisai Gerchen, Peter Kirsch, Sabine Vollstädt-Klein, Falk Kiefer, Patrick Bach, Bernd Lenz

**Affiliations:** ^1^Department of Addictive Behavior and Addiction Medicine, Central Institute of Mental Health, Medical Faculty Mannheim, Heidelberg University, Mannheim, Germany; ^2^Department of Biostatistics, Central Institute of Mental Health, Medical Faculty Mannheim, Heidelberg University, Mannheim, Germany; ^3^Department of Clinical Psychology, Central Institute of Mental Health, Medical Faculty Mannheim, Heidelberg University, Mannheim, Germany; ^4^Bernstein Center for Computational Neuroscience Heidelberg-Mannheim, Mannheim, Germany; ^5^Department of Psychology, Heidelberg University, Heidelberg, Germany; ^6^Mannheim Center for Translational Neurosciences, Medical Faculty Mannheim, Heidelberg University, Mannheim, Germany; ^7^Feuerlein Center on Translational Addiction Medicine, Heidelberg University, Heidelberg, Germany

**Keywords:** sex difference, alcohol cue reactivity, alcohol use disorder, addiction, craving, fMRI

## Abstract

**Background:**

Males consume more alcohol than females, and alcohol use disorder (AUD) is more prevalent in males than females. However, females progress faster to AUD. Sex differences in neural alcohol cue reactivity were previously observed in young social drinkers, indicating a role of hypersensitivity to alcohol-related cues in very early stages of addiction. To our knowledge, this is the first study on patients diagnosed with AUD to test sex differences in neural reactivity to alcohol cues in order to widen previous findings.

**Methods:**

We analyzed data from previous studies, using a well-established functional magnetic resonance imaging (fMRI) paradigm to compare neural reactivity to alcohol cues between 42 female and 124 male patients with AUD (mean age 45 and 46 years) in predefined regions of interest that were implicated by previous studies (ventral and dorsal striatum as well as caudate, putamen, amygdala, hippocampus, insula, anterior cingulate cortex, and medial prefrontal cortex) using independent samples *t*-tests. Post-hoc, effect size calculations were performed.

**Results:**

Throughout all nine regions of interest, we found no statistically significant sex differences in neural reactivity toward alcoholic pictures alone or in comparison to neutral pictures (*p* > 0.05, FDR-corrected). Post-hoc effect size estimates indicated a magnitude between 0.137 and 0.418 (Hedge’s *g*) on alcohol reactivity to alcohol cues compared to neutral cues and indicate very small to less than medium effect sizes in the direction of higher cue reactivity in female patients.

**Conclusion:**

Previous studies showed sex differences in neural alcohol cue reactivity in younger social and problematic alcohol drinkers, i.e., stronger striatal cue-reactivity in males. After correction for multiple comparisons, we did not observe significant sex differences in a cohort of middle-aged females and males with AUD. Sex differences that are present during early phases of addiction development might disappear at later stages of AUD and might thus be considered as clinically less relevant in patients with more severe AUD.

## Introduction

Alcohol is responsible for 3 million deaths and 132.6 million disability-adjusted life years (DALYs) per year worldwide, which accounts for 5.3% of global deaths and 5.1% of DALYs [estimate of the World Health Organization for 2016 (WHO, 2018)]. Sex differences regarding alcohol consumption and alcohol use disorder (AUD) are widely accepted and can be explained by biological and psychosocial factors (Ceylan-Isik et al., 2010; Becker et al., 2017). In Europe and the Americas, males drink more alcohol than females and are 2 to 4 times more prone to AUD (Rehm et al., 2015; WHO, 2018). By contrast, females show an accelerated progression from initiation of alcohol use to AUD (Zilberman et al., 2004) and a higher vulnerability to the sequelae of chronic alcohol intake such as liver damage (Agabio et al., 2017). Whereas males with AUD drink alcohol more often to facilitate social interaction, females use it more frequently to regulate symptoms of anxiety and depression or stress (Peltier et al., 2019; Müller et al., 2021). Further, female compared to male patients with AUD were observed to report generally stronger substance craving (Boykoff et al., 2010), they also showed a stronger relation between craving and negative affect throughout the detoxification process (Petit et al., 2017). However, recent observations indicate a closing gap with respect to sex differences in alcohol consumption and related consequences (White et al., 2015; Slade et al., 2016; White, 2020).

A stronger neural cue reactivity relates to negative outcomes of substance consumption, such as addiction severity, substance craving, or treatment outcomes, and the underlying brain regions include circuits associated with reward, motivation, and goal directed behavior, as well as habit learning (Kühn and Gallinat, 2011; Jasinska et al., 2014). Specifically, meta-analyses have shown that alcohol cues induce activation of subcortical and prefrontal brain regions, i.e., ventral striatum (VS), anterior cingulate cortex (ACC), and ventromedial prefrontal cortex (vmPFC) (Schacht et al., 2013). To our knowledge, only two functional magnetic resonance imaging (fMRI) studies have been published so far to report on sex-differing effects of neural alcohol cue reactivity (Seo et al., 2011; Kaag et al., 2019). During brief guided imagery, male social drinkers (*N* = 20, mean age 32.5 years) showed stronger stress-related activation than female social drinkers (*N* = 23, mean age 30.9 years) in the medial prefrontal cortex, rostral anterior cingulate cortex, posterior insula, amygdala, and hippocampus and weaker alcohol cue-reactivity in the superior and middle frontal gyrus (Seo et al., 2011). During a visual alcohol cue task, alcohol compared to neutral stimuli elicited stronger reactivity of the VS and dorsal striatum (DS) with medium to large effect sizes (VS η_*p*_^2^ = 0.10, DS η_*p*_^2^ = 0.11) in males (*N* = 28, mean age 26.0 years) compared to females (*N* = 27, mean age 24.9 years) with problematic alcohol consumption (Kaag et al., 2019). Notably, substance use, anxiety, and/or depressed mood did not account for the sex effects on reactivity of the DS. Taken together, the existing literature on sex differences in neuronal reactivity to alcohol cues is limited. Only small cohorts with rather young and non-dependent subjects have been investigated. A better understanding of sex differences in alcohol cue reactivity will inform the development of personalized strategies to treat patients with AUD, i.e., sex-sensitive prevention, diagnosis, and treatment approaches (Lenz et al., 2012; Barker and Taylor, 2019).

This study aimed to identify sex differences in brain reactivity to visual alcohol cues within specific regions of interest (ROI) in a larger sample of male and female patients in treatment for AUD. Based on the existing literature (Kaag et al., 2019), we hypothesized stronger alcohol cue reactivity in the dorsal striatum (DS) and ventral striatum (VS) in males compared to females. Based on previously reported findings and as corticostriatal-limbic regions are relevant in AUD (Zeng et al., 2021), we further examined sex differences in alcohol cue reactivity in the ACC, the medial prefrontal cortex (medPFC), the insula, the amygdala, and the hippocampus (including a core circuit for emotion regulation, Ochsner et al., 2004).

## Materials and methods

### Procedure and participants

Between 2008 and 2015, treatment-seeking patients with AUD from the Department of Addictive Behavior and Addiction Medicine, Central Institute of Mental Health (CIMH), Mannheim, Germany, participated in one of three studies, respectively, previously conducted in our lab (e.g., Vollstädt-Klein et al., 2011, 2012; Kiefer et al., 2015; Bach et al., 2020, 2022). Baseline functional magnetic resonance imaging (fMRI) examination prior to any study intervention was used for the here presented analyses [*N* = 178 (23.6% female)]. The diagnosis of an alcohol dependency, corresponding to a moderate to severe AUD, was made by trained medical staff following the International Classification of Diseases (ICD-10). A diagnosis of alcohol dependency following the ICD-10 or Diagnostic and Statistical Manual of Mental Disorders, 4th edition (DSM-IV) can be considered as moderate to severe AUD according to the DSM, 5th edition (DSM-5) (American Psychiatric Association, 2013; Hasin et al., 2013; Hoffmann and Kopak, 2015). All study participants were given full particulars about the study and open questions were answered. Subsequently, all participants provided written informed consent prior to study participation. The local Ethics Committee II of the Medical Faculty Mannheim, Heidelberg University, approved the corresponding studies (approval number study 1 + 2: 2007-095F-MA; study 3: 2011-303-MA).

Following study inclusion, a structured clinical interview was conducted to examine possible comorbid mental disorders (Wittchen et al., 1997). Individuals with substance use disorder besides alcohol and nicotine or comorbid mental disorders within the last 12 months, neurological or severe somatic conditions, a history of brain injury, a use of psychotropic substances or medication (except during detoxification), and contraindications for an MRI examination were excluded from study participation. Prior to scanning, participants were free from any detoxification medication for at least 3 days, abstained from alcohol for 3−21 days, and did not show withdrawal symptoms [measured with the Clinical Institute Withdrawal Assessment scale (CIWA-Ar), Sullivan et al., 1989].

Before proceeding with the fMRI data acquisition, sociodemographic information was collected and participants completed self-report questionnaires. Alcohol craving and AUD severity were examined using the Obsessive-Compulsive Drinking Scale-German (OCDS-G; Anton, 2000; Mann and Ackermann, 2000) and the Alcohol Dependence Scale (ADS; Skinner and Horn, 1984; Doyle and Donovan, 2009), respectively. The last drinking day prior to the fMRI examination was assessed, and the Beck Depression Inventory (BDI-II; Beck et al., 1961), the trait questions from the State-Trait Anxiety Inventory (STAI; Laux et al., 1981), and the Fagerstroem Test for Nicotine Dependence (FTND; Heatherton et al., 1991) were applied.

### Magnetic resonance imaging data acquisition

#### Functional magnetic resonance imaging paradigm

A well-established fMRI paradigm was used to examine neural alcohol cue reactivity (Vollstädt-Klein et al., 2010, 2012). In brief, alcohol-related (beer, wine, and schnapps) and neutral stimuli were presented in pseudo-randomized blocks. Slight changes in the application of the paradigm arose due to the combined data from three different studies. In study 1 and study 2, participants were asked to evaluate their alcohol craving following each alcohol-related or neutral block of stimuli (Vollstädt-Klein et al., 2010; studies 1 and 2, *N* = 113). In study 3, pictures were only passively viewed, and craving was not assessed between alcohol-related or neutral blocks of stimuli (Bach et al., 2020; *N* = 65).

#### Functional magnetic resonance imaging acquisition

Scanning was performed using a 3T whole-body tomograph (MAGNETOM Trio, TiM-technology; Siemens, Erlangen, Germany). During all studies, images were presented to patients using MRI Audio/Video Systems goggles (Resonance Technology Inc., Los Angeles, CA, United States). The paradigm was presented using Presentation^®^ software (Neurobehavioral Systems, Inc., Albany, CA, United States). For each subject, T2*-weighted echo-planar images (EPI) were acquired in a transversal orientation 30°clockwise to the bicommissural line (AC-PC line) using the same parameters for all three studies [repetition time (TR) = 2.41 s, echo time (TE) = 25 ms, flip angle = 80°, 42 slices, slice thickness: 2 mm, 1 mm gap, voxel size 3 × 3 × 3 mm^3^, field of view (FOV) 192 × 192 mm^2^, 64 × 64 in-plane resolution]. Additionally, a 5:21 min anatomical scan was performed to acquire a T1-weighted 3D MPRAGE (Magnetization Prepared-Rapid Gradient Echo) dataset [192 sagittal slices, TR = 2.30 s, TE = 3.03 ms, inversion time (TI) = 900 ms, flip angle = 9°, slice thickness: 1 mm, 0.5 mm gap, voxel dimensions 1 × 1 × 1.5 mm^3^, FOV 256 × 256 mm^2^, 256 × 256 in-plane resolution].

### Data analysis

Pre-processing and individual statistical analyses of brain imaging data were performed using SPM12 (Wellcome Centre for Human Neuroimaging, London, United Kingdom). Subsequent descriptive and statistical analyses were performed in SPSS (IBM SPSS Statistics for Windows, Version 27.0, IBM Corp., Armonk, NY, United States).

#### Functional magnetic resonance imaging data preprocessing

For each functional data set, the first five scans were excluded from the analyses to avoid artifacts caused by magnetic saturation effects. The remaining scans were spatially realigned to correct for head motion, temporally realigned to minimize temporal differences in slice acquisition, and normalized to the SPM12 tissue probability map template provided by MNI (Montreal Neurological Institute, Montreal, QC, Canada). Subsequent smoothing of the images was performed using an isotropic Gaussian kernel (6 mm full width at half maximum, FWHM).

#### First-level functional magnetic resonance imaging analyses

Statistical analyses of the pre-processed fMRI data on the first-level (single subject) were performed by modeling the contrasts for alcohol-related stimuli (“alcohol” to implicit baseline), neutral stimuli (“neutral” to implicit baseline), and “alcohol > neutral,” i.e., neural reactivity during the presentation of alcohol-related stimuli vs. neutral stimuli. A general linear model (GLM) on a voxel-by-voxel basis was applied and convolution with the canonical hemodynamic response function was used. A high-pass filter was used (cut-off at 128 s). All six motion parameters from the preprocessing were included as regressors of no interest. A quality check was performed and subjects with excessive head movement (>3 mm/3°) or other artifacts were excluded from the subsequent analysis.

#### Anatomical masks and regions of interest data extraction

Preselected ROI are based on previous research on neural correlates of cue reactivity, cognitive control, and reward processing in AUD (Ochsner et al., 2004; Seo et al., 2011; Schacht et al., 2013; Kaag et al., 2019). The anatomical masks for the left and right hemispheres separately were created using the human WFU_pickatlas v3^1^. The implemented aal (anatomical automatic labeling) atlas was used for mean data extraction of the ACC, medPFC (superior medial PFC), amygdala, and hippocampus. The implemented IBASPM71 atlas was used for mean data extraction of the caudate, insula, and putamen. Following Kaag et al. (2019), the VS was defined as the nucleus accumbens (NAcc) and the DS was defined as a combined mask of the putamen and caudate (i1) minus the ventral striatum (i2) using the ImCalc function in SPM12 (i1–i2). In total, nine bilateral ROI were created. Data extraction of parameter estimates was performed for the individual contrasts “alcohol,” “neutral,” and “alcohol > neutral” within each of the aforementioned ROI. An automatized script based on SPM12 was used for extracting and averaging the data (mean) per ROI.

#### Analysis of sociodemographic, psychometric, and regions of interest data

Group characteristics with respect to study and sex were examined and are reported in the Supplementary material S1. Regarding ROI data, extreme outliers (values more than three box lengths from either hinge) were defined following the inspection of boxplots and the computation of inter quartile ranges in SPSS and excluded from subsequent analyses if they appeared for the contrasts “alcohol” or “neutral” (*N* = 12). *N* = 166 data sets (42 females) were available for subsequent analyses.

Variables that could potentially influence neural cue reactivity were examined (days of abstinence, smoking status, age; see Supplementary material S2). Subsequently, independent samples *t*-tests for continuous data or chi-square tests for dichotomous data were calculated to test for sex differences. Correction for multiple comparisons was applied using the false discovery rate (FDR) with the Benjamini and Hochberg method (Storey, 2002). Exploratory analyses to examine the influence of craving and severity of AUD on sex effects were conducted. Univariate GLM were used to assess the influence of sex as well as craving for alcohol and severity of AUD on neural cue reactivity within the nine predefined ROI for the contrasts “alcohol,” “neutral,” and “alcohol > neutral.” Uncorrected results are reported in the following. As for both, craving and severity of AUD, no interaction effects with sex emerged, these terms were removed from the analyses and only main effects are reported (Supplementary Tables S2, S3). All analyses included age as a potentially confounding variable.

Lastly, independent samples *t*-tests were used to test for sex differences with respect to the category of beverage (beer, wine, and schnapps) and are reported in the Supplementary material S3.

#### Effect size calculations

As for effect size (ES) calculations based on the previous analyses, Hedges’ *g* was estimated in SPSS. Post-hoc power analyses and sample size calculations using the here observed ES were conducted using G*Power 3.1 (Faul et al., 2007).

In order to generate further exploratory value from our analyses, additional voxel-wise calculations of ES and corresponding confidence intervals were performed post-hoc. The procedure follows Gerchen et al. (2021). In short, whole brain two-sample *t*-tests were calculated in SPM12 for the contrast “alcohol > neutral” for “males > females.” Using the resulting t-maps and applying the “g_es_ci” Matlab script (Gerchen et al., 2021), the standardized ES Hedges’ *g* and the corresponding 95-% confidence intervals (CI) were calculated for all voxels in the nine ROI separately. Subsequently all ROI masks were resized to the same MNI dimensions as the ES g-map. Finally and separately for each of the aforementioned ROI, an in-house Matlab script was used to firstly create a data matrix including the ES as well as the lower and upper bound of the corresponding CI per voxel; secondly sort the data from low to high ES values; thirdly plot the distribution of ES including the lower and upper CI; and fourthly extract the median ES for each ROI (see Supplementary Figure S1).

## Results

### Sample characteristics

After exclusion of individuals with extreme outliers (*N* = 12), the study sample consisted of *N* = 166 individuals with AUD (25% females, 68% smokers). No significant difference in the sex ratio (male vs. female) emerged when comparing the three studies [χ^2^(2) = 2.621, *p* = 0.270]. No significant sex differences were observed for the severity of dependence, craving, days of abstinence, and further variables (*p* > 0.05), see Table 1. Results from analysis examining these variables with respect to all three studies can be found in the Supplementary material S1.

**TABLE 1:** Sample description.

	Males *M* (*SD*) or %	Females *M* (*SD*) or %	Statistics	*p*
*N*	124 (74.7%)	42 (25.3%)	χ^2^(2) = 2.621	*p* = 0.270^a^
Age (years)	46.3 (10.3)	45.3 (8.8)	*t*(164) = 0.577	*p* = 0.565
Living (%; alone:together with others)	49:51	32:68	χ^2^(1) = 3.629	*p* = 0.057
Education (years)	13.6 (2.8)	13.7 (2.7)	*t*(162) = −0.213	*p* = 0.832
Smoker (%; yes:no)	69:31	67:33	χ^2^(1) = 0.051	*p* = 0.821
FTND (sum score, for smokers only)	5.3 (2.7)	5.2 (2.7)	*t*(111) = 0.272	*p* = 0.768
DSM-5 criteria	6.0 (1.1)	5.8 (1.5)	*t*(133) = 1.129	*p* = 0.261
Days of abstinence	11.3 (10.8)	12.1 (11.2)	*t*(156) = −0.373	*p* = 0.710
ADS (sum score)	15.8 (7.0)	15.9 (6.6)	*t*(161) = −0.106	*p* = 0.916
OCDS-G (sum score)	20.5 (8.0)	20.3 (7.6)	*t*(162) = 0.114	*p* = 0.909
STAI (trait sum score)	45.1 (11.9)	46.5 (9.8)	*t*(158) = −0.658	*p* = 0.512
BDI-II (sum score)	13.4 (9.8)	11.7 (8.4)	*t*(162) = 0.647	*p* = 0.336

Sex differences in sociodemographic and clinical variables are displayed. M, Mean; SD, Standard Deviation; *N*, sample size. ADS, Alcohol Dependence Scale; BDI-II, Beck Depression Inventory II; DSM-5, Diagnostic and Statistical Manual of Mental Disorders, 5th edition (available for studies 2 and 3); FTND, Fagerstroem Test for Nicotine Dependence; OCDS-G, Obsessive-Compulsive Drinking Scale-German (global score); STAI, State-Trait-Anxiety Inventory. Two-sided *t*-tests and chi-square tests were used to address group differences. Results of *t*-tests are reported following the assessment of homogeneity of variances. Uncorrected *p*-values are displayed. ^a^Comparison of sex ratio (females vs. males) between all three studies.

### Neural cue reactivity

Following independent samples *t*-tests and FDR correction for multiple testing, no significant sex differences regarding the contrasts “alcohol,” “neutral,” or “alcohol > neutral” were observed for the DS and VS (main hypothesis) or additional ROI (exploratory hypothesis) (*p* > 0.05) (see Tables 2A–C). Post-hoc analyses revealed that the sample sizes of the present data sets were not sufficient for detecting the corresponding effects with a power of ≥0.80 and an alpha error probability of ≤0.05.

**TABLE 2:** Independent samples *t*-tests for all nine regions of interest for the contrasts “alcohol”, “neutral”, and “alcohol > neutral”.

(A) “alcohol”										

	Males *M* (*SD*)	Females *M* (*SD*)	Statistic	*p*	*p*_*FDR–corrr*_	mean_DIFF_ (SE_DIFF_)	95%-CI lower| upper	*g*	1-β	*N*_req_ ^a^
*N*	124	42								
DS	0.002 (0.373)	−0.012 (0.364)	*t*(164) = 0.210	0.834	0.938	0.014 (0.066)	−0.117 | 0.145	0.037	0.055	22,936
VS	0.067 (0.440)	0.139 (0.431)	*t*(164) = −0.917	0.360	0.878	−0.072 (0.078)	−0.226 | 0.083	−0.163	0.149	1,184
Putamen	0.159 (0.332)	0.187 (0.335)	*t*(164) = −0.472	0.638	0.878	−0.028 (0.059)	−0.145 | 0.089	−0.084	0.075	4,452
Caudate	−0.169 (0.563)	−0.229 (0.531)	*t*(164) = 0.605	0.546	0.878	0.060 (0.099)	−0.136 | 0.256	0.107	0.092	2,746
Amygdala	0.398 (0.476)	0.396 (0.603)	*t*(164) = 0.027	0.978	0.978	0.003 (0.091)	−0.178 | 0.183	0.005	0.050	1,255,820
Insula	0.082 (0.420)	0.136 (0.375)	*t*(164) = −0.739	0.461	0.878	−0.054 (0.073)	−0.198 | 0.090	−0.131	0.113	1,832
Hippocampus	0.327 (0.440)	0.372 (0.358)	*t*(164) = −0.596	0.552	0.878	−0.045 (0.075)	−0.193 | 0.104	−0.106	0.091	2,798
medPFC	0.024 (0.602)	0.156 (0.635)	*t*(164) = −1.212	0.227	0.878	−0.132 (0.109)	−0.347 | 0.083	−0.215	0.224	682
ACC	−0.121 (0.429)	−0.090 (0.399)	*t*(164) = −0.409	0.683	0.878	−0.031 (0.075)	−0.179 | 0.118	−0.073	0.069	5,894

**(B) “neutral”**										

*N*	124	42								
DS	−0.023 (0.171)	−0.073 (0.147)	*t*(164) = 1.697	0.092	0.318	0.050 (0.029)	−0.008 | 0.108	0.302	0.391	348
VS	−0.019 (0.166)	−0.029 (0.171)	*t*(164) = 0.351	0.726	0.726	0.010 (0.030)	−0.048 | 0.069	0.062	0.065	8,170
Putamen	0.048 (0.136)	0.010 (0.115)	*t*(164) = 1.628	0.106	0.318	0.038 (0.023)	−0.008 | 0.084	0.289	0.363	378
Caudate	−0.100 (0.267)	−0.163 (0.239)	*t*(164) = 1.356	0.177	0.398	0.063 (0.046)	−0.029 | 0.155	0.241	0.268	544
Amygdala	0.148 (0.221)	0.080 (0.210)	*t*(164) = 1.749	0.082	0.318	0.068 (0.039)	−0.009 | 0.145	0.311	0.410	328
Insula	0.015 (0.193)	−0.013 (0.148)	*t*(164) = 0.838	0.403	0.452	0.027 (0.033)	−0.037 | 0.092	0.149	0.132	1,418
Hippocampus	0.117 (0.186)	0.086 (0.145)	*t*(164) = 0.985	0.326	0.452	0.031 (0.032)	−0.031 | 0.093	0.175	0.164	1,028
medPFC	−0.074 (0.250)	−0.114 (0.239)	*t*(164) = 0.897	0.371	0.452	0.040 (0.044)	−0.048 | 0.127	0.159	0.143	1,244
ACC	−0.117 (0.188)	−0.147 (0.183)	*t*(164) = 0.905	0.367	0.452	0.030 (0.033)	−0.036 | 0.096	0.161	0.146	1,214

**(C) “alcohol > neutral”**										

*N*	124	42								
DS	0.053 (0.343)	0.152 (0.334)	*t*(164) = −1.622	0.107	0.216	−0.099 (0.061)	−0.219 | 0.021	−0.288	0.361	382
VS	0.065 (0.414)	0.160 (0.409)	*t*(164) = −1.291	0.198	0.235	−0.095 (0.074)	−0.241 | 0.050	−0.229	0.247	602
Putamen	0.029 (0.286)	0.152 (0.313)	*t*(164) = −2.350	0.020	0.180	−0.123 (0.052)	−0.226 | −0.020	−0.418	0.643	182
Caudate	0.080 (0.547)	0.152 (0.423)	*t*(164) = −0.773	0.440	0.440	−0.072 (0.093)	−0.254 | 0.111	−0.137	0.119	1,676
Amygdala	0.061 (0.471)	0.197 (0.526)	*t*(164) = −1.565	0.120	0.216	−0.135 (0.087)	−0.306 | 0.035	−0.278	0.340	410
Insula	0.036 (0.424)	0.149 (0.471)	*t*(164) = −1.445	0.150	0.225	−0.112 (0.078)	−0.266 | 0.041	−0.257	0.299	478
Hippocampus	0.055 (0.431)	0.174 (0.324)	*t*(164) = −1.636	0.104	0.216	−0.119 (0.073)	−0.262 | 0.025	−0.291	0.367	374
medPFC	0.164 (0.697)	0.381 (0.544)	*t*(164) = −1.842	0.067	0.216	−0.218 (0.118)	−0.451 | 0.016	−0.327	0.445	296
ACC	0.132 (0.469)	0.236 (0.430)	*t*(164) = −1.263	0.209	0.235	−0.104 (0.082)	−0.265 | 0.058	−0.224	0.239	628

Mean values of cue reactivity (standard deviation), corresponding statistics, and Hedge’s *g* are displayed. Actual power and required sample size for an equal male:female ratio were calculated. M, Mean; SD, Standard Deviation; SE, Standard Error; *N*, sample size. *P*_FDR–corr_, corrected *p*-value using the False Discovery Rate; DIFF, Difference; 95%-CI, 95% Confidence Interval; *g*, Hedge’s *g*; 1-β, Power; *N*_req_, required sample size to detect the calculated effect with a power of 0.80, an alpha error probability of 0.05, and equal allocation ratio of males and females in an two-sided independent *t*-test; DS, Dorsal Striatum; VS, Ventral Striatum (Nucleus Accumbens); medPFC, superior medial Prefrontal Cortex; ACC, Anterior Cingulate Cortex. Two-sided *t*-tests were used to test for group differences between males and females. Results of *t*-tests are reported following the assessment of homogeneity of variances. Corrections of *p*-values using the False Discovery Rate were conducted per contrast and according to Storey (2002). Power analyses and hypothetical sample sizes were calculated using G*Power (Faul et al., 2007) using the estimated effect size (Hedge’s *g*) as calculated in SPSS. ^a^sample size with a 1:1 ratio of male:female.

The additional ES calculation (based on the effect size calculation for each voxel as described before) resulted in median ES for the contrast males > females between −0.093 (Caudate) and −0.247 (Putamen). As depicted in Supplementary Figure S1 the lack of significant group differences emerged once again.

#### Exploratory analyses of further sex effects

Post-hoc exploratory analyses were conducted to examine the possibly interacting influence of craving or severity of dependence on sex-effects in order to broaden our understanding of the present data.

When only adjusting for age, a significant sex effect was observed for the contrast “alcohol > neutral” on putamen activity (*F*(1, 166) = 5.666, *p* = 0.018, partial η^2^ = 0.034; males-females: MeanDiff = −0.125 (0.052), 95%-CI [−0.228, −0.021]). No further significant sex effects were observed.

When adjusting for craving (OCDS-G) and age, no significant interaction of sex and severity of craving was observed, and the interaction term was removed for subsequent analyses. An effect of sex was observed on neural activity of the putamen and medPFC for “alcohol > neutral” [putamen: *F*(1, 160) = 6.296, *p* = 0.013, partial η^2^ = 0.038; males-females: MeanDiff = −0.133 (0.053), 95%-CI [−0.238, −0.028]; medPFC: *F*(1, 160) = 4.035, *p* = 0.046, partial η^2^ = 0.025; males-females: MeanDiff = −0.241 (0.120), 95%-CI [−0.477, −0.004]; Supplementary Table S2C] and on neural activity of the putamen, DS, and amygdala for “neutral” (see Supplementary Table S2B). No significant sex effects emerged for other contrasts or ROI. OCDS-G score excerpted a significant influence on neural activity for the contrast “alcohol” (putamen, VS, amygdala, and hippocampus, Supplementary Table S2A). Please see Supplementary Table S2 for more details.

When adjusting for AUD severity (ADS) and age, no significant interaction of sex and severity of AUD was observed, and the interaction term was removed for subsequent analyses. A significant effect of sex effect was observed on neural reactivity of the putamen and medPFC for “alcohol > neutral” [putamen: *F*(1, 159) = 6.306, *p* = 0.013, partial η^2^ = 0.038; males-females: MeanDiff = −0.134 (0.052), 95%-CI [−0.240, −0.029]; medPFC: *F*(1, 159) = 4.145, *p* = 0.043, partial η^2^ = 0.025; males-females: MeanDiff = −0.244 (0.120), 95%-CI [−0.482, −0.007]; see Supplementary Table S3C] and on neural activity of the putamen, DS, and amygdala for “neutral” (see Supplementary Table S3B). No significant sex effects emerged for other contrasts or ROI. ADS score did not excerpt a significant influence on neural activity. Please see Supplementary Table S3 for more details.

Further, sex differences in neural reactivity with regard to specific alcoholic beverages (i.e., beer, wine, and schnapps) were examined as they might present a sex-specific bias. No statistically significant group differences were observed (p_FDRcorr_ > 0.05). Analyses procedure and results are reported in the Supplementary material S3.

Whole-brain analyses on sex-differences in neural alcohol cue-reactivity for the contrast “alcohol > neutral” are reported in the Supplementary material S4. Males compared to females showed significantly less activation within the bilateral superior and middle occipital gyri, left lingual and fusiform gyri as well as the precentral and inferior frontal gyri, and right cuneus (family wise error-corrected threshold of *p* < 0.05).

## Discussion

The aim of the study was to identify sex differences in neural reactivity toward alcohol stimuli of brain regions relevant for AUD in a sample of treatment-seeking in-patients with AUD. Here, we did not observe the hypothesized significantly higher alcohol cue reactivity in striatal regions in males vs. females. Remarkably, in the VS we did not even identify a single voxel with an effect in the hypothesized direction. We further did not observe significant sex differences in other brain regions commonly involved. On a descriptive level, in our data all ROI showed a tendency toward larger cue reactivity in females with ES on averaged data ranging from −0.137 to −0.418 (Table 2C) and median voxel-wise ES ranging from −0.093 to −0.247 (Supplementary Figure S1) for the task contrast “alcohol > neutral.”

Examining humans and rodents, prenatal and adult sex hormone activities have been related to alcohol consumption, brain structure, and neural function (Hermans et al., 2010; Lenz et al., 2012, 2017, 2018; Huber et al., 2018; Siegmann et al., 2019). Previous studies showed sex-specific neural reactivity toward alcohol cues with stronger effects in the superior and middle frontal gyrus in women with social alcohol drinking (Seo et al., 2011) and stronger striatal reactivity in men with problematic alcohol consumption (Kaag et al., 2019). Further, female-specific estradiol effects to induce dorsolateral striatal drug response have been observed in cocaine-dependent rats (Cummings et al., 2014). In nicotine-dependent women, neural reactivity of the putamen to smoking compared to non-smoking cues has further been shown to be dependent on the menstrual cycle status (Franklin et al., 2019). Interestingly, our uncorrected results (Table 2C) suggest that women with AUD might show stronger putamen reactivity toward substance cues - even though the direction of the effect opposes our hypothesis.

Several factors might explain the absence of the expected sex differences in our cohort.

*First*, considering both previous studies and our analyses, we studies individuals with AUD compared to social or heavy drinkers. Our data might indicate that sex differences in alcohol cue reactivity disappear from early to later phases of AUD development (see for e.g., Kaag et al., 2019). The incentive salience of alcohol stimuli represents a major contributor to alcohol use during early AUD, and more habit-associated mechanisms become relevant during later stages, i.e., severe AUD. Indeed, striatal neural reactivity toward alcohol cues has been suggested to be contingent on several factors. A shift from VS to DS cue reactivity from heavy social drinking to alcohol dependence (Vollstädt-Klein et al., 2010) and increased reactivity of the putamen during the first 2 weeks of abstinence (Bach et al., 2020) have been observed.

*Second*, previous studies including smaller sample sizes might have over-estimated ES. Our study was sufficiently powered to detect sex differences with medium ES. However, the Hedges’ g ES of the sex differences observed here varied between | 0.005| for the “alcohol” contrast in the amygdala and | 0.418| for the “alcohol > neutral” contrast in the putamen, which can be considered as very small to small. Further and with respect to a power of 0.80, it is possible that medium size effects might have been missed, even though they truly exist. To detect the maximal effect that we identified with this power, an even larger sample size of *N* = 182 (91 males and 91 females) would have been necessary.

When using a slightly different approach (Gerchen et al., 2021), voxel-wise ES estimation as well as corresponding CI were calculated and depicted. Besides the visual representation of the Hedges’ g ES per voxel within one ROI, displaying the CI allows for assessing the range of values that the ES of a specific voxel lies within – in our analyses with a probability of 95%. This procedure further allows for navigating the uncertainty of how well this sample of *N* = 166 individuals with AUD estimates the ES within a specific ROI for comparing alcohol vs. neutral cues when assessing sex differences, i.e., narrow CIs represent precise estimations. We could also, for example, inspect the figure with a reference of *g* = 0.2 (small effect) to evaluate the question of how many voxel within a specific ROI might have a probable ES superior to this or use an ES interval to examine the number of voxel within this range. The position of the ES curve with respect to null of the *Y*-axis (ES) also indicates a bias within the overall data distribution and can be quantified using the median effect size as a characteristic value of this ROI. Importantly, our analyses and visual presentation of the data does not only result in a deeper understanding of the lack of significant sex effects, as compared to solely stating a *p*-value. It further serves the community regarding the planning of the sample size and direction of effect (rather females > males) for future, confirmatory studies. However, our results suggest that possible sex effects are rather of small size. Thus, they could be of limited clinical relevance and might not significantly contribute to improving therapy outcomes. Even though sex differences were observed previously, e.g., on an endocrine level, they might not be strongly relevant in terms of a pathological neurobiological phenotype.

*Third*, differences in age might account for the divergence of the previous and our current findings. Our study population was older (45.4 and 46.1 years for females and males) than the cohorts of Kaag et al. (2019) (24.9 and 26.0 years) and Seo et al. (2011) (30.9 and 32.5 years). However and after adjusting for age, there were also no sex effects on neural cue reactivity of the VS or DS.

Sex differences in the development, maintenance, and phenomenology of AUD have to be acknowledged in research and treatment, and differences in alcohol drinking between men and women decline (Mccaul et al., 2019; White, 2020). Even though females are more at risk for severe somatic sequelae of alcohol, they are, compared to males, underrepresented in treatment settings for AUD (Mccaul et al., 2019). However, we also did not observe significant interaction of main effects of sex with either severity of craving or AUD. The uncorrected results indicate that craving influences neural cue reactivity toward alcoholic stimuli in both male and female patients with AUD as we observed that alcohol craving excerpted an influence on neural reactivity toward alcoholic stimuli in the putamen, VS, amygdala, and hippocampus (see Supplementary Table S2). The relation between self-reported craving and neural cue reactivity is well documented for alcohol and other substances (Jasinska et al., 2014).

Several limitations have to be discussed. The sample was unevenly balanced with respect to males and females. As it consisted of treatment-seeking patient, one cannot exclude that individual patients already have acquired and possibly used craving regulation strategies during the experiment. Patients were not instructed to respond in any specific way to the presented stimuli but were asked to rate their craving between blocks of alcohol and neutral stimuli in studies 1 and 2, but not study 3. Not giving specific instruction with regard to how to look at the stimuli [e.g., (not) regulating one’s craving] might also contribute to the here presented findings. Also, time since the last cigarette was not assessed but represents a major factor influencing alcohol cue-reactivity. It should further be noted that certain aspects, typically observed in female study participants, were not addressed in the current analyses due to the original study design, especially hormonal medication and menstrual cycle status. Both are of importance since oral contraceptives exert hormonal effects on the central nervous system (Hampson, 2020), mesolimbic incentive processing changes during the menstrual cycle (Dreher et al., 2007), and alterations in estradiol and progesterone activities associate with AUD and related phenotypes (Mühle et al., 2019; Weinland et al., 2021). In a monetary incentive delay task, women show stronger ventral striatal activation during the premenstrual than during the follicular phase (Ossewaarde et al., 2011). Also, a premenstrual volume increase of the amygdala has been reported and correlated with higher stress-induced negative affect (Ossewaarde et al., 2013). Cue reactivity in striatal regions, such as the putamen, were found to be modulated by menstrual cycle status in cigarette smokers (Franklin et al., 2019). Moreover, we did not correct for diurnal variations in craving (West and Schneider, 1987).

Consequently, future studies are necessary to investigate neural cue reactivity in a longitudinal design to address the question, whether prominent sex differences in neural cue reactivity diminishes over time and whether the small effect of increased cue reactivity in females might be more pronounced when specific phases of the menstrual cycle are analyzed separately. In terms of losing and regaining control over alcohol consumption, the causal association between female-specific aspects such as hormonal fluctuations and the progression from social drinking to severe AUD, as well as examining the complex interplay between sex and further (clinical) factors are subject to future research. Addressing the hypotheses of stronger (striatal) alcohol cue-reactivity in females when examining underlying causalities for the faster progression from drinking to being alcohol-dependent might open new perspectives that might then be translated into sex- and gender-oriented addiction therapy.

## Conclusion

In this study, we did not observe significant sex differences in neural alcohol cue reactivity in patients with AUD. We further found only very small to less than medium effect sizes in the direction of higher cue reactivity in female patients. Previous studies in younger social and problematic alcohol drinkers indicate sex differences in neural cue reactivity within reward-related brain regions (males > females). Women might differ from men in terms of neural reactivity to alcohol cues during early phases of the addiction development. However, our data indicates that a clinically significant effect of sex differences might diminish in later phases of AUD.

## Data availability statement

The original contributions presented in this study are included in the article/Supplementary material, further inquiries can be directed to the corresponding author.

## Ethics statement

The studies involving human participants were reviewed and approved by the Local Ethics Committee II of the Medical Faculty Mannheim, Heidelberg University, Mannheim, Germany (approval number study 1 + 2: 2007-095F-MA; study 3: 2011-303-MA). The patients/participants provided their written informed consent to participate in this study.

## Author contributions

SG, PB, and BL designed the current study. SG analyzed the data and wrote the manuscript. SH, HT, MG, SV-K, and PB supported the data analysis. PB and BL supported to write the manuscript. SG, SH, PB, and BL interpreted the data. SV-K, PK, and FK conceived and designed the original experiments. PK and FK procured the funding of the original studies. All authors commented on the manuscript and provided intellectual input.

## Footnotes

1
https://www.nitrc.org/projects/wfu_pickatlas/
