# Supplementary material for: Neural cue reactivity is not stronger in male than in female patients with alcohol use disorder
Source: Front Behav Neurosci. 2022 Nov 16;16:1039917. doi: 10.3389/fnbeh.2022.1039917 (PMC9709253; doi:10.3389/fnbeh.2022.1039917)

**Supplementary Material S1**

**Merging of studies for subsequent analyses**

Prior to merging imaging and clinical data from all three studies, group characteristics, i.e., sex ratio, age, days of abstinence, alcohol craving (Obsessive-Compulsive Drinking Scale - German Version, OCDS-G), severity of alcohol use disorder (AUD) (Alcohol Dependence Scale, ADS), depressiveness (Beck Depression Inventory II, BDI-II), anxiety (State-Trait-Anxiety Inventory, STAI trait), and smoking status with respect to study and sex were examined using independent samples *t*-tests for continuous data or chi-square tests for dichotomous data. When appropriate, Games-Howell post-hoc tests were conducted.

No significant differences with respect to study and sex emerged for age, smoking status (smoker vs. non-smoker), Fagerstroem Test for Nicotine Dependence (FTND) sum score (smokers only), ADS sum score, or STAI trait sum score (*p* > 0.05). However, craving significantly differed between studies (OCDS-G *F*(2, 157) = 20.586, *p* < 0.001) for the overall sample, and both males and females separately (males *F*(2, 122) = 12.191, *p* < 0.001, females *F*(2, 25.20) = 12.148, *p* < 0.001). Post-hoc test indicated significant differences between studies 1 and 3 and between studies and 2 and 3, respectively: significantly lower sum scores of the OCDS-G questionnaire were observed for the passive viewing study (study 3) compared to studies 1 and 2. Regarding separate analyses for males and females, males reported significant less years of education for study 1 compared to study 3 (*F*(2,56.4) = 5.526, *p* = 0.006). Regarding separate analyses for studies 1, 2, and 3, males reported significantly less years of education in study 1 (*t*(27) = -5.483, *p* = 0.027). Uncorrected statistical results are reported.

**Supplementary Material S2**

**Confounding variables**

Possibly confounding variables influencing neural cue reactivity were examined. Neither days of abstinence nor smoking status revealed significant results for all aforementioned regions of interest (ROI) (partial correlations controlling for sex between days of abstinence and mean activation within ROI, *p* > 0.05; independent samples t-tests between smokers and non-smokers, *p* > 0.05). However, significant results emerged for the partial correlation (controlling for sex) between age and mean activation within some (i.e., putamen, caudate, DS) but not all ROI (see Supplementary Table S1). Age did not significantly correlate with either craving (OCDS-G *r* = -0.021, *p*_uncorr_ = 0.796) or severity of alcohol dependence (AD) (ADS *r* = -0.142, *p*_uncorr_ = 0.078).

**Supplementary Material S3**

**Exploratory analyses of sex differences with regard to types of beverage**

To examine possible sex-differences with respect to the type of alcoholic stimulus presented, data extraction of parameter estimates was performed for the individual contrasts ‘beer’, ‘wine’, and ‘schnapps’ for participants with available data regarding these contrasts (*N* = 113 from study 1 and study 2; 27 females, 86 males). Using two sample *t*-tests to examine sex differences in neural cue reactivity to specific beverage within each of the nine ROI, no statistically significant group differences were observed following the same analyses procedure as for the main analyses in the manuscript (*p*_FDRcorr_ > 0.05).

**Supplementary Material S4**

**Exploratory whole-brain analyses of sex differences**

To examine whole-brain sex-differences regarding the contrast ‘alcohol > neutral’, an independent samples *t*-test was performed in SPM12. The same data set (*N* = 166 individuals) as for the main analyses was used and age was included as a covariate of no interest. Using a family-wise error (FWE)-corrected threshold of *p* < 0.001 in combination with a cluster-extend threshold 183 voxel of (following the random field theory in SPM), three significant clusters emerged for the group comparison. Males exhibited less activation compared to females when viewing alcohol compared to neutral pictures. The three clusters encompassed voxels located in the bilateral superior and middle occipital gyri, left lingual and fusiform gyri as well as precentral and inferior frontal gyri, and right cuneus. Please see Supplementary Table S4 and Supplementary Figure S2 for more details.

**SUPPLEMENTARY TABLES**

**Supplementary Table S1: Correlations between age and ROI activation while controlling for sex.**

|  | **DS** | **VS** | **Putamen** | **Caudate** | **Amygdala** | **Insula** | **Hippocampus** | **medPFC** | **ACC** |
| --- | --- | --- | --- | --- | --- | --- | --- | --- | --- |
| **‘alcohol’** | -0.215** | -0.005 | -0.114 | -0.225** | - 0.116 | 0.008 | -0.088 | 0.022 | 0.004 |
| **‘neutral’** | -0.258*** | -0.093 | -0.180* | -0.243** | -0.051 | -0.128 | -0.059 | -0.139 | -0.152 |
| **‘alcohol > neutral’** | 0.028 | 0.065 | 0.062 | 0.001 | -0.086 | 0.129 | -0.024 | 0.118 | 0.139 |

**Note**. DS, Dorsal Striatum; VS, Ventral Striatum (Nucleus Accumbens); medPFC, superior medial Prefrontal Cortex; ACC, Anterior Cingulate Cortex. Correlation coefficients (r) are displayed and uncorrected *p*-values are reported. **p* < 0.05, ***p* < 0.01, ****p* < 0.001

**Supplementary Table S2: General linear model to examine the sex effect on neural cue-reactivity. Craving and age were included as covariates.** Separate models were calculated for different ROI and contrasts. Significant results are highlighted in bold.

| A – ‘alcohol’ |  | |  |  |  |  |  |  |  |  |  |  |  |
| --- | --- | --- | --- | --- | --- | --- | --- | --- | --- | --- | --- | --- | --- |
|  | **Males**  **M (SD)** | | **Females**  **M (SD)** | **Statistic**  **sex** | ***p*** | **η^2^** | **B** | **95% CI** | **Statistic**  **OCDS-G** | ***p*** | **η^2^** | **B** | **95% CI** |
| *N* | 123 | | 41 |  |  |  |  |  |  |  |  |  |  |
| DS | 0.002 (0.375) | | -0.017 (0.372) | F(1,160) = 0.147 | 0.702 | 0.001 | 0.025 | -0.105 \| 0.156 | F(1,160) = 0.271 | 0.603 | 0.002 | 0.002 | -0.005 \| 0.009 |
| VS | 0.070 (0.441) | | 0.146 (0.434) | F(1,160) = 0.977 | 0.324 | 0.006 | -0.078 | -0.233 \| 0.077 | **F(1,160) = 5.229** | **0.024** | **0.032** | **0.010** | **0.001 \| 0.018** |
| Putamen | 0.160 (0.333) | | 0.189 (0.333) | F(1,160) = 0.203 | 0.653 | 0.001 | -0.027 | -0.144 \| 0.091 | **F(1,160) = 4.766** | **0.030** | **0.029** | **0.007** | **0.001 \| 0.014** |
| Caudate | -0.171 (0.565) | | -0.243 (0.530) | F(1,160) = 0.704 | 0.403 | 0.004 | 0.083 | -0.112 \| 0.277 | F(1,160) = 0.499 | 0.481 | 0.003 | -0.004 | -0.015 \| 0.007 |
| Amygdala | 0.402 (0.477) | | 0.396 (0.610) | F(1,160) = 0.008 | 0.928 | 0.000 | 0.008 | -0.171 \| 0.188 | **F(1,160) = 5.835** | **0.017** | **0.035** | **0.012** | **0.002 \| 0.022** |
| Insula | 0.084 (0.421) | | 0.136 (0.380) | F(1,160) = 0.526 | 0.469 | 0.003 | -0.054 | -0.200 \| 0.093 | F(1,160) = 2.785 | 0.100 | 0.017 | 0.007 | -0.001 \| 0.015 |
| Hippocampus | 0.329 (0.442) | | 0.375 (0.362) | F(1,160) = 0.369 | 0.544 | 0.002 | -0.045 | -0.193 \| 0.102 | **F(1,160) = 9.009** | **0.003** | **0.053** | **0.012** | **0.004 \| 0.020** |
| medPFC | 0.022 (0.604) | | 0.160 (0.642) | F(1,160) = 1.578 | 0.211 | 0.010 | -0.140 | -0.360 \| 0.609 | F(1,160) = 0.106 | 0.745 | 0.001 | 0.002 | -0.010 \| 0.014 |
| ACC | -0.121 (0.431) | | -0.092 (0.404) | F(1,160) = 0.139 | 0.710 | 0.001 | -0.029 | -0.181 \| 0.123 | F(1,160) = 0.328 | 0.568 | 0.002 | -0.002 | -0.011 \| 0.006 |
| B – ‘neutral’ |  | |  |  |  |  |  |  |  |  |  |  |  |
|  | **Males**  **M (SD)** | | **Females**  **M (SD)** | **Statistic**  **sex** | ***p*** | **η^2^** | **B** | **95% CI** | **Statistic**  **OCDS-G** | ***p*** | **η^2^** | **B** | **95% CI** |
| *N* | 123 | | 41 |  |  |  |  |  |  |  |  |  |  |
| DS | -0.023 (0.171) | | -0.084 (0.130) | **F(1,160) = 5.034** | **0.026** | **0.031** | **0.064** | **0.008 \| 0.120** | F(1,160) = 0.424 | 0.516 | 0.003 | 0.001 | -0.002 \| 0.004 |
| VS | -0.019 (0.166) | | -0.031 (0.173) | F(1,160) = 0.205 | 0.651 | 0.001 | 0.014 | -0.046 \| 0.073 | F(1,160) = 2.142 | 0.145 | 0.013 | 0.002 | -0.001 \| 0.006 |
| Putamen | 0.049 (0.136) | | 0.003 (0.108) | **F(1,160) = 4.186** | **0.042** | **0.025** | **0.047** | **0.002 \| 0.093** | F(1,160) = 0.771 | 0.381 | 0.005 | -0.002 | -0.001 \| 0.004 |
| Caudate | -0.102 (0.267) | | -0.179 (0.218) | F(1,160) = 3.289 | 0.072 | 0.020 | 0.082 | -0.007 \| 0.171 | F(1,160) = 0.134 | 0.715 | 0.001 | 0.001 | -0.004 \| 0.006 |
| Amygdala | 0.150 (0.220) | | 0.069 (0.200) | **F(1,160) = 4.424** | **0.037** | **0.027** | **0.082** | **0.005 \| 0.159** | F(1,160) = 0.003 | 0.957 | 0.000 | 0.000 | -0.004 \| 0.004 |
| Insula | 0.017 (0.192) | | -0.021 (0.140) | F(1,160) = 1.459 | 0.229 | 0.009 | 0.039 | -0.025 \| 0.103 | F(1,160) = 0.300 | 0.585 | 0.002 | 0.001 | -0.003 \| 0.005 |
| Hippocampus | 0.118 (0.186) | | 0.076 (0.134) | F(1,160) = 1.809 | 0.181 | 0.011 | 0.042 | -0.020 \| 0.105 | F(1,160) = 0.186 | 0.667 | 0.001 | 0.001 | -0.003 \| 0.004 |
| medPFC | -0.074 (0.251) | | -0.122 (0.236) | F(1,160) = 1.253 | 0.265 | 0.008 | 0.050 | -0.038 \| 0.137 | F(1,160) = 0.613 | 0.435 | 0.004 | -0.003 | -0.007 \| 0.001 |
| ACC | -0.116 (0.188) | | -0.155 (0.178) | F(1,160) = 1.553 | 0.214 | 0.010 | 0.042 | -0.024 \| 0.107 | F(1,160) = 0.008 | 0.930 | 0.000 | 0.000 | -0.004 \| 0.003 |
| C – ‘alcohol > neutral’ | |  |  |  |  |  |  |  |  |  |  |  |  |
|  | **Males**  **M (SD)** | | **Females**  **M (SD)** | **Statistic**  **sex** | ***p*** | **η^2^** | **B** | **95% CI** | **Statistic**  **OCDS-G** | ***p*** | **η^2^** | **B** | **95% CI** |
| *N* | 123 | | 41 |  |  |  |  |  |  |  |  |  |  |
| DS | 0.054 (0.322) | | 0.160 (0.334) | F(1,160) = 2.997 | 0.085 | 0.018 | -0.107 | -0.229 \| 0.015 | F(1,160) = 0.772 | 0.381 | 0.005 | 0.003 | -0.004 \| 0.010 |
| VS | 0.067 (0.415) | | 0.169 (0.411) | F(1,160) = 2.015 | 0.158 | 0.012 | -0.105 | -0.252 \| 0.041 | F(1,160) = 3.521 | 0.062 | 0.022 | 0.008 | 0.000 \| 0.016 |
| Putamen | 0.029 (0.287) | | 0.160 (0.313) | **F(1,160) = 6.296** | **0.013** | **0.038** | **-0.133** | **-0.238 \|-0.028** | F(1,160) = 1.106 | 0.295 | 0.004 | 0.003 | -0.003 \| 0.006 |
| Caudate | 0.081 (0.549) | | 0.159 (0.426) | F(1,160) = 0.686 | 0.409 | 0.004 | -0.078 | -0.265 \| 0.108 | F(1,160) = 0.317 | 0.574 | 0.002 | 0.003 | -0.007 \| 0.013 |
| Amygdala | 0.061 (0.473) | | 0.205 (0.529) | F(1,160) = 2.592 | 0.109 | 0.016 | -0.142 | -0.315 \| 0.032 | F(1,160) = 0.700 | 0.404 | 0.004 | 0.004 | -0.006 \| 0.014 |
| Insula | 0.036 (0.425) | | 0.157 (0.473) | F(1,160) = 2.608 | 0.108 | 0.016 | -0.127 | -0.282 \| 0.028 | F(1,160) = 1.511 | 0.221 | 0.009 | 0.005 | -0.003 \| 0.014 |
| Hippocampus | 0.054 (0.433) | | 0.184 (0.322) | F(1,160) = 3.076 | 0.081 | 0.019 | -0.129 | -0.275 \| 0.016 | F(1,160) = 1.332 | 0.250 | 0.008 | 0.005 | -0.003 \| 0.013 |
| medPFC | 0.162 (0.699) | | 0.396 (0.543) | **F(1,160) = 4.035** | **0.046** | **0.025** | **-0.241** | **-0.477 \|-0.004** | F(1,160) = 0.094 | 0.760 | 0.001 | 0.002 | -0,011 \| 0.095 |
| ACC | 0.132 (0.470) | | 0.246 (0.462) | F(1,160) = 2.099 | 0.149 | 0.013 | -0.120 | -0.283 \| 0.044 | F(1,160) = 0.700 | 0.404 | 0.004 | 0.004 | -0.005 \| 0.013 |

**Note**: M, Mean; SD, Standard Deviation; η^2^, eta square; B, beta coefficient; CI, confidence interval; *N*, sample size. OCDS-G, German version of the Obsessive-Compulsive Drinking Scale. DS, Dorsal Striatum; VS, Ventral Striatum (Nucleus Accumbens); medPFC, superior medial Prefrontal Cortex; ACC, Anterior Cingulate Cortex. Uncorrected *p* values are displayed.

**Supplementary Table S3: General linear model to examine the sex effect on neural cue-reactivity. Severity of alcohol use disorder and age were included as covariates.** Separate models were calculated for different ROI and contrasts. Significant results are highlighted in bold.

| A – ‘alcohol’ |  | |  |  |  |  |  |  |  |  |  |  |  |
| --- | --- | --- | --- | --- | --- | --- | --- | --- | --- | --- | --- | --- | --- |
|  | **Males**  **M (SD)** | | **Females**  **M (SD)** | **Statistic**  **sex** | ***p*** | **η^2^** | **B** | **95% CI** | **Statistic**  **ADS** | ***p*** | **η^2^** | **B** | **95% CI** |
| *N* | 122 | | 41 |  |  |  |  |  |  |  |  |  |  |
| DS | -0.001 (0.374) | | -0.017 (0.367) | F(1,159) = 0.109 | 0.742 | 0.001 | 0.022 | -0.108 \| 0.152 | F(1,159) = 0.663 | 0.417 | 0.004 | -0.003 | -0.005 \| 0.004 |
| VS | 0.067 (0.448) | | 0.146 (0.434) | F(1,159) = 0.973 | 0.325 | 0.006 | -0.078 | -0.235 \| 0.079 | F(1,159) = 1.871 | 0.173 | 0.012 | -0.007 | -0.017 \| 0.003 |
| Putamen | 0.158 (0.333) | | 0.189 (0.339) | F(1,159) = 0.210 | 0.647 | 0.001 | -0.028 | -0.147 \| 0.092 | F(1,159) = 0.186 | 0.667 | 0.001 | -0.002 | -0.009 \| 0.001 |
| Caudate | -0.176 (0.565) | | -0.243 (0.530) | F(1,159) = 0.600 | 0.440 | 0.004 | 0.076 | -0.118 \| 0.270 | F(1,159) = 0.739 | 0.391 | 0.005 | -0.005 | -0.018 \| 0.007 |
| Amygdala | 0.403 (0.478) | | 0.396 (0.610) | F(1,159) = 0.016 | 0.910 | 0.000 | 0.012 | -0.172 \| 0.195 | F(1,159) = 0.028 | 0.866 | 0.000 | 0.001 | -0.011 \| 0.013 |
| Insula | 0.080 (0.421) | | 0.136 (0.380) | F(1,159) = 0.553 | 0.458 | 0.003 | -0.056 | -0.203 \| 0.092 | F(1,159) = 0.009 | 0.924 | 0.000 | 0.000 | -0.009 \| 0.010 |
| Hippocampus | 0.329 (0.444) | | 0.375 (0.362) | F(1,159) = 0.315 | 0.576 | 0.002 | -0.043 | -0.194 \| 0.108 | F(1,159) = 1.965 | 0.163 | 0.012 | 0.007 | -0.003 \| 0.017 |
| medPFC | 0.016 (0.602) | | 0.160 (0.642) | F(1,159) = 1.696 | 0.195 | 0.011 | -0.145 | -0.365 \| 0.075 | F(1,159) = 0.138 | 0.711 | 0.001 | 0.003 | -0.011 \| 0.017 |
| ACC | -0.127 (0.426) | | -0.092 (0.404) | F(1,159) = 0.218 | 0.641 | 0.001 | -0.036 | -0.186 \| 0.115 | F(1,159) = 0.495 | 0.483 | 0.003 | -0.003 | -0.013 \| 0.006 |
| B – ‘neutral’ |  | |  |  |  |  |  |  |  |  |  |  |  |
|  | **Males**  **M (SD)** | | **Females**  **M (SD)** | **Statistic**  **sex** | ***p*** | **η^2^** | **B** | **95% CI** | **Statistic**  **ADS** | ***p*** | **η^2^** | **B** | **95% CI** |
| *N* | 122 | | 41 |  |  |  |  |  |  |  |  |  |  |
| DS | -0.023 (0.172) | | -0.084 (0.130) | **F(1,159) = 5.002** | **0.027** | **0.031** | **0.064** | **0.007 \| 0.120** | F(1,159) = 0.299 | 0.585 | 0.002 | 0.001 | -0.003 \| 0.002 |
| VS | -0.020 (0.166) | | -0.031 (0.173) | F(1,159) = 0.162 | 0.688 | 0.001 | 0.012 | -0.047 \| 0.072 | F(1,159) = 0.968 | 0.327 | 0.006 | -0.002 | -0.006 \| 0.006 |
| Putamen | 0.048 (0.136) | | 0.003 (0.108) | **F(1,159) = 4.021** | **0.047** | **0.025** | **0.121** | **0.001 \| 0.092** | F(1,159) = 0.190 | 0.663 | 0.001 | -0.001 | -0.004\| 0.002 |
| Caudate | -0.101 (0.268) | | -0.179 (0.218) | F(1,159) = 3.367 | 0.068 | 0.021 | 0.083 | -0.006 \| 0.172 | F(1,159) = 0.953 | 0.330 | 0.006 | 0.003 | -0.003 \| 0.009 |
| Amygdala | 0.151 (0.221) | | 0.069 (0.218) | **F(1,159) = 4.447** | **0.037** | **0.027** | **0.083** | **0.005 \| 0.160** | F(1,159) = 0.001 | 0.980 | 0.000 | 0.000 | -0.005 \| 0.005 |
| Insula | 0.015 (0.192) | | -0.201 (0.140) | F(1,159) = 1.366 | 0.244 | 0.009 | 0.038 | -0.026 \| 0.102 | F(1,159) = 1.172 | 0.281 | 0.007 | -0.038 | -0.026 \| 0.102 |
| Hippocampus | 0.120 (0.186) | | 0.076 (0.134) | F(1,159) = 1.898 | 0.128 | 0.015 | 0.044 | -0.019 \| 0.106 | F(1,159) = 0.113 | 0.737 | 0.001 | 0.001 | -0.000 \| 0.005 |
| medPFC | -0.075 (0.252) | | -0.122 (0.236) | F(1,159) = 1.219 | 0.271 | 0.008 | 0.049 | -0.039 \| 0.138 | F(1,159) = 0.009 | 0.926 | 0.000 | 0.000 | -0.005 \| 0.006 |
| ACC | -0.118 (0.188) | | -0.155 (0.185) | F(1,159) = 1.419 | 0.235 | 0.009 | 0.039 | -0.026 \| 0.105 | F(1,159) = 1.466 | 0.228 | 0.009 | -0.003 | -0.007 \| 0.002 |
| C – ‘alcohol > neutral’ | |  |  |  |  |  |  |  |  |  |  |  |  |
|  | **Males**  **M (SD)** | | **Females**  **M (SD)** | **Statistic**  **sex** | ***p*** | **η^2^** | **B** | **95% CI** | **Statistic**  **ADS** | ***p*** | **η^2^** | **B** | **95% CI** |
| *N* | 122 | | 41 |  |  |  |  |  |  |  |  |  |  |
| DS | 0.050 (0.344) | | 0.160 (0.334) | F(1,159) = 3.151 | 0.078 | 0.019 | -0.110 | -0.232 \| 0.012 | F(1,159) = 0.934 | 0.335 | 0.006 | -0.004 | -0.012 \| 0.004 |
| VS | 0.066 (0.417) | | 0.169 (0.411) | F(1,159) = 1.920 | 0.168 | 0.012 | -0.104 | -0.253 \| 0.044 | F(1,159) = 0.000 | 0.983 | 0.000 | 0.000 | -0.010 \| 0.009 |
| Putamen | 0.027 (0.288) | | 0.160 (0.313) | **F(1,159) = 6.306** | **0.013** | **0.038** | **-0.134** | **-0.240 \|-0.029** | F(1,159) = 0.001 | 0.980 | 0.000 | 0.000 | -0.007 \| 0.007 |
| Caudate | 0.077 (0.548) | | 0.159 (0.526) | F(1,159) = 0.776 | 0.380 | 0.005 | -0.083 | -0.269 \| 0.103 | F(1,159) = 1.836 | 0.177 | 0.011 | -0.008 | -0.038 \| 0.007 |
| Amygdala | 0.060 (0.474) | | 0.205 (0.529) | F(1,159) = 2.561 | 0.112 | 0.016 | -0.142 | -0.316 \| 0.033 | F(1,159) = 0.022 | 0.881 | 0.000 | 0.001 | -0.010 \| 0.012 |
| Insula | 0.033 (0.426) | | 0.157 (0.473) | F(1,159) = 2.605 | 0.108 | 0.016 | -0.127 | -0.283 \| 0.028 | F(1,159) = 1.074 | 0.302 | 0.007 | 0.005 | -0.005 \| 0.015 |
| Hippocampus | 0.052 (0.434) | | 0.184 (0.322) | F(1,159) = 3.104 | 0.080 | 0.019 | -0.131 | -0.277 \| 0.016 | F(1,159) = 0.348 | 0.556 | 0.002 | 0.003 | -0.007 \| 0.012 |
| medPFC | 0.157 (0.700) | | 0.396 (0.543) | **F(1,159) = 4.145** | **0.043** | **0.025** | **-0.244** | **-0.482 \|-0.007** | F(1,159) = 0.018 | 0.893 | 0.000 | -0.001 | -0.016 \| 0.014 |
| ACC | 0.128 (0.470) | | 0.246 (0.430) | F(1,159) = 2.168 | 0.143 | 0.013 | -0.122 | -0.286 \| 0.042 | F(1,159) = 0.021 | 0.884 | 0.000 | 0.001 | -0.010 \| 0.011 |

M, Mean; SD, Standard Deviation; η^2^, eta square; B, beta coefficient; CI, confidence interval; *N* = sample size. ADS, Alcohol Dependence Scale; DS, Dorsal Striatum; VS, Ventral Striatum (Nucleus Accumbens); medPFC, superior medial Prefrontal Cortex; ACC, Anterior Cingulate Cortex. Uncorrected *p* values are displayed.

**Supplementary Table S4:** Following a whole-brain analyses, an independent samples *t*-test including age as a covariate revealed hypoactivation in males compared to females for the contrast ‘alcohol > neutral’.

| **Side** | **Lobe** | **Brain Areas** | **Brodmann Area** | **Cluster Size** | **MNI Coordinates** | | ***t*_max_** |
| --- | --- | --- | --- | --- | --- | --- | --- |
| Left | Occipital | Middle and superior occipital gyrus, lingual gyrus, fusiform gyrus | 18, 19 | 790 | -16 -98 14 | | 5.081 |
| Right | Occipital | Middle and superior occipital gyrus, cuneus | 18, 19 | 226 | 14 -88 34 |  | 4.783 |
| Left | Frontal | Inferior frontal gyrus, precentral gyrus | 9 | 193 | -42 12 34 |  | 4.354 |
| **Note:** MNI, Montreal Neurological Institute. A combined voxel-wise- [*p* < 0.001] and cluster-extent threshold [*k* >= 183 voxel] following the random-field theory in SPM12 was applied, corresponding to *p*FWE < 0.05. | | | | | | | |

**SUPPLEMENTARY FIGURES**

**Supplementary Figure S1: Sorted Hedges’ g effect sizes (blue lines) as well as their upper and lower confidence intervals (red lines) of all voxels within each of the nine ROI.** The underlying effect for calculation the effect sizes and confidence intervals is the comparison ‘males > females’ for the contrast ‘alcohol > neutral’ . Median effect sizes are as follows: Dorsal Striatum = -0.168, Ventral Striatum = -0.158, Putamen = -0.247, Caudate = -0.093, Amygdala = -0.224, Insula = -0.146, Hippocampus = -0.167, medPFC (Medial Superior Frontal region) = -0.205, ACC (Anterior Cingulate Cortex) = -0.135. A reference line at g = 0 as well as grid lines are displayed for easing interpretation of the data, e.g., superiority or inferiority.


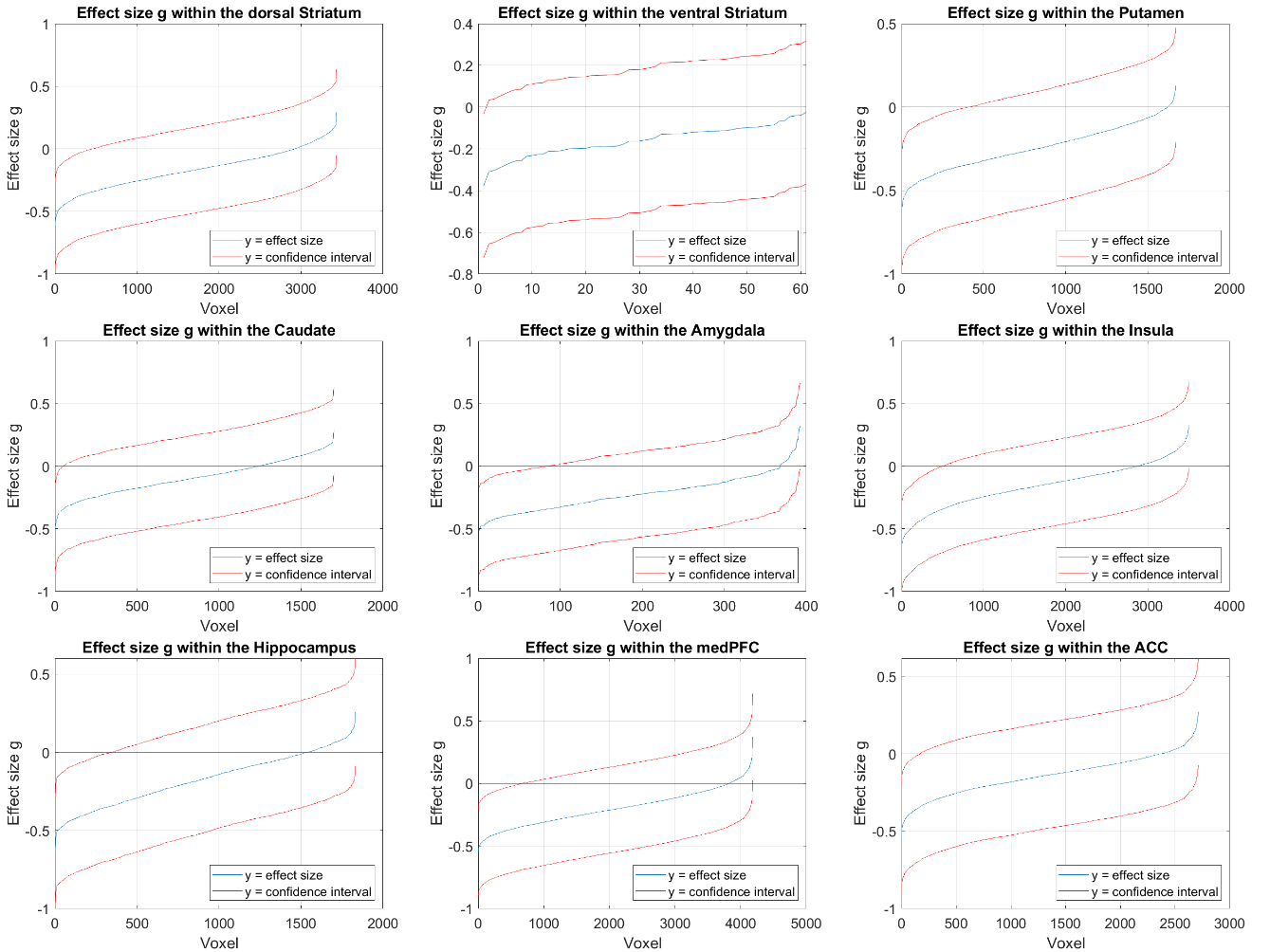


**Supplementary Figure S2: Whole-brain analyses of sex-differences in alcohol-cue reactivity.** An independent samples *t*-test including age as a covariate revealed hypoactivation in males compared to females for the contrast ‘alcohol > neutral’ in the (A) precentral and inferior frontal gyri (x y z, -42 12 34) as well as the (B) bilateral superior and middle occipital gyri, left lingual and fusiform gyri (x y z, -16 -98 14) (family-wise error-corrected threshold of *p* < 0.05).


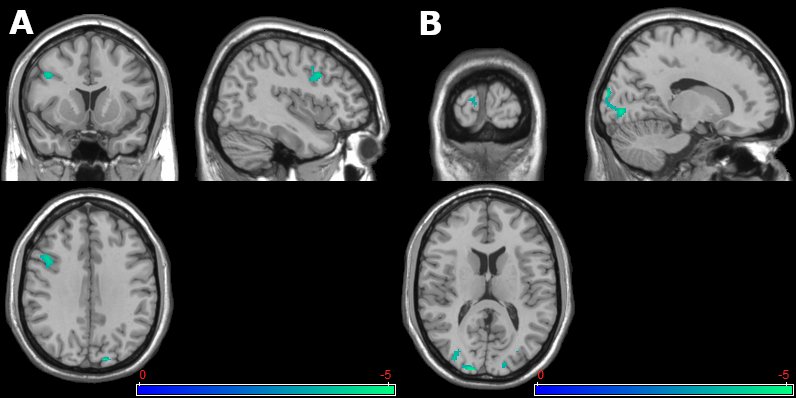

Supplement: Supplementary file 1 [file Data_Sheet_1.docx]
